# Prognostic Value of Serum Albumin in Aortic Aneurysm Patients Undergoing Graft Replacement of Ascending Aorta and Aortic Arch

**DOI:** 10.7150/ijms.81884

**Published:** 2023-04-02

**Authors:** Won Seok Nam, Myung Il Bae, Sang Beom Nam, Suk Won Song, Arim Jo, Sung Yeon Ham

**Affiliations:** 1 Department of Anesthesiology and Pain Medicine, Yonsei University College of Medicine, Seoul, South Korea; 2 Anesthesia and Pain Research Institute, Yonsei University College of Medicine, Seoul, South Korea; 3 Department of Thoracic and Cardiovascular Surgery, Yonsei University College of Medicine, Seoul, South Korea

**Keywords:** Hypoalbuminemia, prognosis, thoracic aortic aneurysm, graft replacement, mortality

## Abstract

**Background:** Hypoalbuminemia is a marker of poor overall health with influences from protein energy malnutrition, systemic inflammation and hepatic and renal disease. Albumin has been reported to have a prognostic impact in various cohorts. This study investigated whether preoperative serum albumin levels could be used to predict mortality in patients with aortic aneurysms undergoing graft replacement of ascending aorta and aortic arch.

**Methods:** We retrospectively reviewed 183 patients who underwent graft replacement of ascending aorta and aortic arch between January 2010 and December 2020. The exclusion criteria included patients with traumatic aortic injuries (n=2), previous aortic repair within 6 months (n=2), ruptured aortic aneurysms (n=14), or a lack of preoperative laboratory data or medical records (n=10). The remaining 87% eligible patients were divided into two groups based on their preoperative serum albumin levels. The lower albumin group was defined as having serum albumin levels ≤3.5 g/dL, while the higher albumin group was defined as having albumin levels >3.5 g/dL. The incidence of mortality was compared between the two groups, and a logistic regression analysis was performed to evaluate the predictors of mortality.

**Results:** The incidence of 1-year mortality was higher in the lower albumin group than in the higher albumin group (3.4% vs. 23.5%, p=0.006). The optimal cut-off value of albumin to predict 1-year mortality was 4.0 g/dL (area under the curve 0.885, 95% CI 0.821-0.949, p<0.001), with a sensitivity and specificity of 90.0% and 80.3%, respectively. Preoperative serum albumin levels (OR = 0.116, 95% CI 0.021-0.641, p=0.014) and diabetes mellitus (OR = 5.757, 95% CI 1.018-32.565, p=0.048) remained independent predictors of mortality.

**Conclusion:** Preoperative serum albumin levels were an independent predictor of 1-year mortality after the graft replacement of ascending aorta and aortic arch. These findings underscore that the optimization of patients' nutritional status before surgery may be warranted and should be further explored in this high-risk population.

## Introduction

Patients undergoing graft replacement of ascending aorta and aortic arch are at high risk for poor clinical outcomes due to the nature of the disease, ischemia-reperfusion injury due to cardiopulmonary bypass, and inflammatory response. 1 A graft repair is the gold standard for treating aortic aneurysms, but the mortality rate is 3%, even in high-volume centers. 2 Therefore, several studies have been conducted to identify the prognostic factors for aortic aneurysms. Hypertension, atherosclerosis, dyslipidemia, and smoking have been identified as risk factors. 3-5

Hypoalbuminemia has been reported to be a prognostic marker for malnutrition, systemic inflammation, and hepatic and renal diseases. 6 Previous studies have shown that serum albumin is a prognostic marker for mortality and morbidity among patients with cardiovascular diseases, those receiving transplants, and those undergoing cardiac/non-cardiac surgeries. 6-9

Aortic aneurysms develop through a process called cystic medial degeneration, which is also associated with connective tissue disorders such as Marfan syndrome and Ehlers-Danlos syndrome. In previous studies on the sporadic forms of thoracic aortic aneurysms, inflammatory and immune cells infiltrated the aortic wall, suggesting that the inflammatory pathway may be critical in the development of thoracic aortic aneurysms. 10 Since the inflammatory response plays an important role in the development of aortic aneurysms and nutrition and systemic inflammation play an important role in the prognosis of patients, several studies have been conducted to elucidate the role of albumin as a prognostic factor in these patients. Hypoalbuminemia is associated with poor clinical outcomes in abdominal aortic aneurysm repair and acute aortic dissection. 11,12 However, the prognostic association between patients undergoing graft replacement of ascending aorta and aortic arch and preoperative serum albumin levels has not been fully described.

Therefore, this study aimed to examine whether serum albumin levels could be a prognostic marker of mortality and morbidity in patients undergoing graft replacement of ascending aorta and aortic arch.

## Methods

This study was approved by the Institutional Review Board of Yonsei University Health System, Seoul, Korea (IRB No.3-2022-0367), and the need for informed consent from the patients was waived. All methods and procedures were performed in accordance with the relevant guidelines and regulations. This study was performed in accordance with the tenets of the Declaration of Helsinki.

### Study population

**Figure 1:**
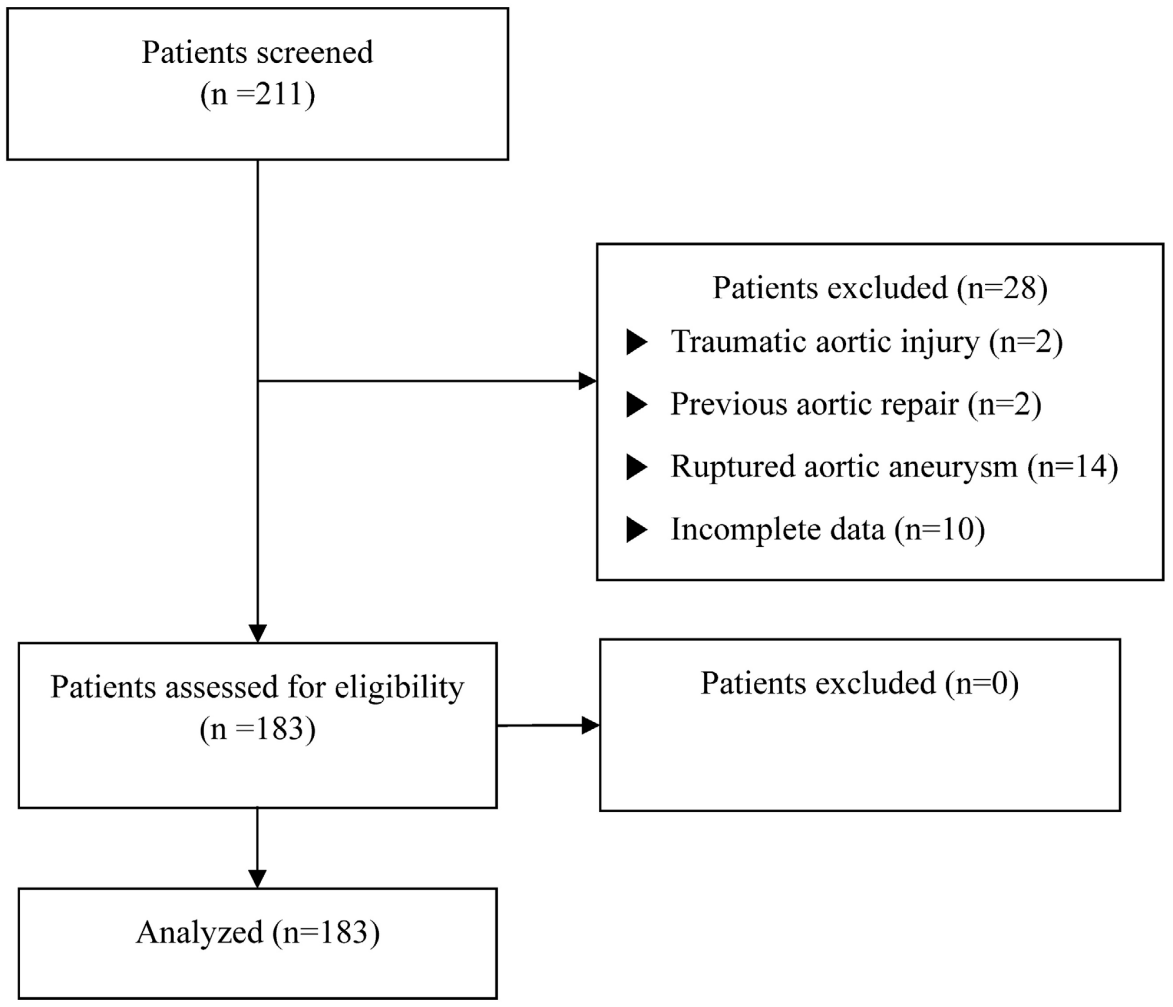
Flowchart of study enrolment

**Figure 2:**
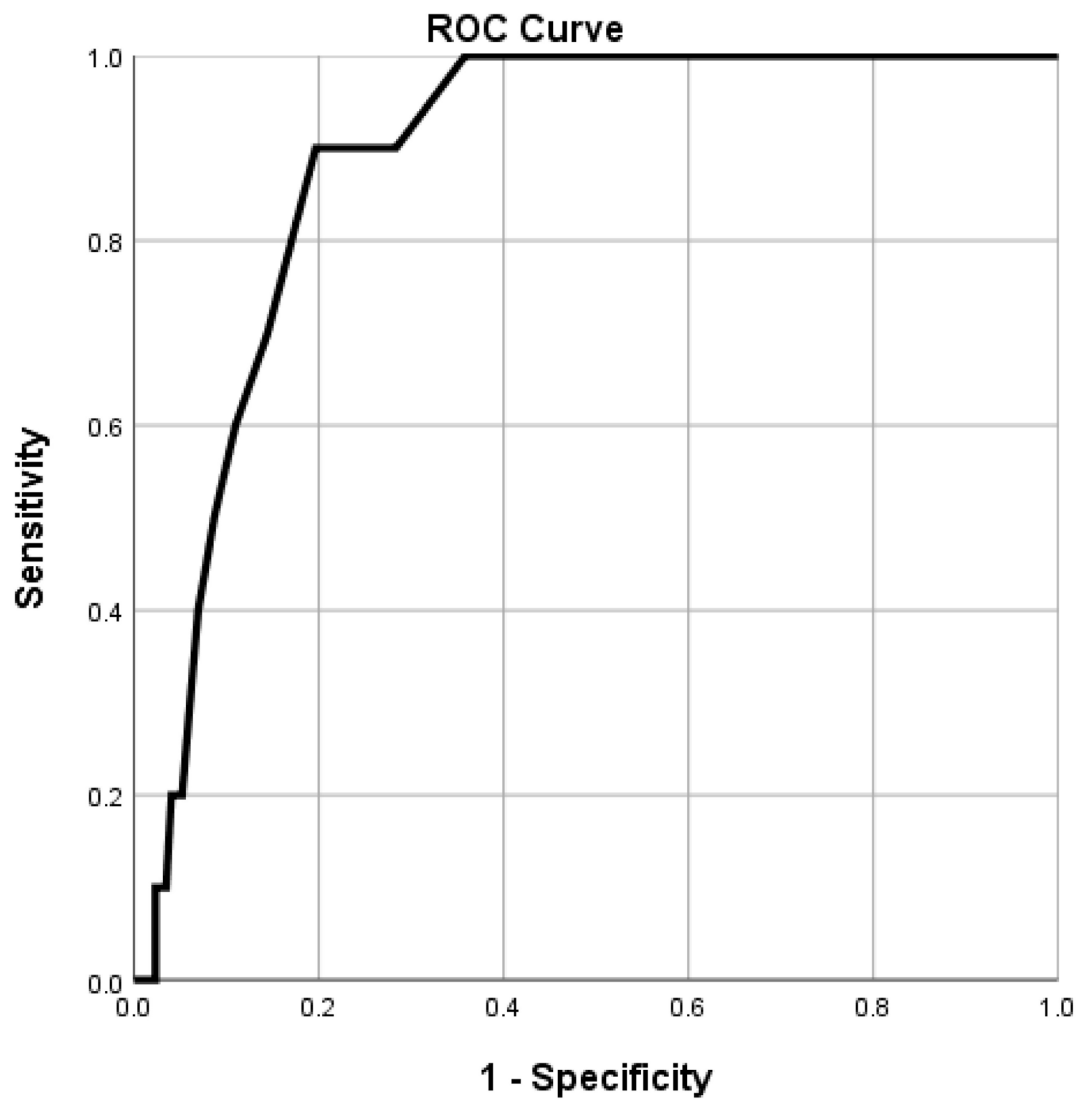
Combined receiver-operating characteristic curve of preoperative albumin levels for the incidence of 1-year mortality. The area under the curve = 0.885 and *p*-values <0.001 are observed below the line showing the serum albumin level with a 95% confidence interval of 0.821-0.949.

We identified all patients who underwent a graft replacement of ascending aorta and aortic arch between January 2010 and December 2020 at the Gangnam Severance Hospital, Yonsei University College of Medicine. Patients with traumatic aortic injuries (n = 2), previous aortic repair within 6 months (n = 2), ruptured aortic aneurysms (n = 14), or a lack of preoperative laboratory data or medical records (n = 10) were excluded. A total of 183 patients were included and analyzed in this study (Figure 1). The patients were divided into two groups based on preoperative serum albumin levels: lower (serum albumin ≤ 3.5 g/dL) and higher (serum albumin > 3.5 g/dL) (Figure 2). 13,14

### Demographic and clinical data

Demographic data included age, sex, height, weight, and current smoking status. Data on medical comorbidities such as hypertension, diabetes mellitus, coronary artery occlusive disease/coronary intervention history, chronic obstructive pulmonary disease, cerebrovascular accident/transient ischemic attack, acute/chronic renal failure, liver disease including fatty liver disease, hepatitis, liver cirrhosis, or hepatocellular carcinoma, and malignancy, and medication (beta-blockers, calcium channel blockers, diuretics, angiotensin receptor blockers) for the mentioned diseases were collected. Perioperative laboratory data (1 month prior to surgery to 1 year postoperatively) included CBC and routine chemistry (serum albumin, CRP, BUN/Cr, eGFR, OT/PT etc.). Postoperative outcomes included postoperative 1-year mortality and morbidities (mechanical ventilation > 24 h, reintubation, wound infection, pulmonary complication, myocardial infarction, arrhythmia, cerebrovascular accident, re-operation, acute kidney injury, use of CRRT). The definition of acute kidney injury was based on the Kidney Disease: Improving Global Outcomes (KDIGO) Clinical Practice Guidelines.

### Study endpoints

The primary endpoint of this study was to determine the prognostic value of preoperative serum albumin levels for predicting mortality in patients undergoing graft replacement of the ascending aorta and aortic arch. The secondary endpoints of this study were postoperative complications, comorbidities (mechanical ventilation > 24 h, reintubation, wound infection, pulmonary complication, myocardial infarction, arrhythmia, cerebrovascular accident, re-operation, acute kidney injury, and use of CRRT), length of hospital stay, and intensive care unit (ICU) stay.

### Statistical analysis

Continuous variables are presented as mean ± standard deviation for normally distributed data, or as medians (interquartile ranges) for skewed data. Normality was assessed using the Kolmogorov-Smirnov test. The independent t-test or Mann-Whitney U test was used to compare continuous variables. Categorical variables are described using absolute and relative (percentage) frequencies. Categorical variables were compared using the chi-squared or Fisher's exact test. We performed a logistic regression analysis to determine the predictors of 1-year mortality. For multivariate analysis, we used a stepwise selection method and selected variables with p < 0.05 in the univariate analysis. Predictability was expressed as odds ratios (OR) and 95% confidence intervals (CI). ROC curve analysis was used to determine the optimal cut-off value of the preoperative serum albumin level showing the best discriminatory capacity to predict postoperative 1-year mortality. Statistical significance was set at p < 0.05. The analysis was performed using SPSS version 23 (IBM Corp., Armonk, NY, USA).

## Results

A total of 211 patients were reviewed during the study period, and 183 patients were analyzed (Figure 1). Patients in the lower albumin group were significantly older than those in the higher albumin group (63.00 [47.00, 71.00] vs. 71.50 [64.00, 77.50], p=0.006). There was a higher prevalence of hypertension CRF (3.6% vs. 18.8%, p=0.034) in the lower albumin group than in the higher albumin group. Preoperative laboratory data including total protein (7.10 [6.80, 7.50]g/dL vs. 5.90 [5.50, 6.40] g/dL, p<0.001), hemoglobin (13.78±1.79 g/dL vs. 10.76±1.72 g/dL, p<0.001), and hematocrit (41.02±5.02 % vs. 32.21±4.57 %, p<0.001) were significantly lower in the lower albumin group than in the higher albumin group. PT (0.99 [0.95, 1.05] sec vs. 1.12 (1.06, 1.24) sec, p<0.001), and CRP (1.00 [0.40, 2.80]mg/L vs. 40.05 [15.05, 78.00] mg/L, p < 0.001) were significantly higher in the lower albumin group than in the higher albumin group. The lower albumin group required more transfusion of packed red blood cells (1.00 [0.00, 2.00] pack vs. 4.00 [1.50, 5.00] pack, p < 0.001) (Table 1).

Table 2 summarizes the incidences of postoperative morbidity and mortality. Hospital day was significantly longer in the lower albumin group (14.00 [11.00, 20.00] day vs. 27.00 [22.00, 50.50] day, p<0.001). The incidence of mechanical ventilation requiring more than 24 h was higher in the lower albumin group than in the higher albumin group (12.6% vs. 43.8%, p=0.004). The postoperative need for continuous renal replacement therapy (3.6% vs. 31.3%, p=0.001) and the incidence of AKI (10.8% vs. 31.3%, p=0.034) were also more common in the lower albumin group than in the higher albumin group. A greater proportion of patients in the lower albumin group had infection (11.4% vs. 43.8%, p=0.003) and pulmonary complications (5.4% vs, 37.5%, p=0.001) than those in the higher albumin group. Moreover, the incidence of 1-year mortality was significantly higher in the lower albumin group than in the higher albumin group (3.6% vs. 25.0%, p=0.006) (Table 2).

The ROC curve of preoperative serum albumin levels for predicting 1-year mortality after graft replacement of ascending aorta and aortic arch demonstrated an area under the curve of 0.885. (95% CI 0.821-0.949, p<0.001). The optimal cut-off value of albumin that predicted the incidence of 1-year mortality was 4.0 g/dL, with a sensitivity and specificity of 90.0% and 80.3%, respectively (Figure 2).

Logistic regression analysis showed that age, diabetes mellitus, preoperative hemoglobin, and preoperative serum albumin level showed a difference with p<0.05 for predicting 1-year mortality of patients. Preoperative serum albumin levels (OR = 0.116, 95% CI 0.021-0.641, p=0.014) and diabetes mellitus (OR = 5.757, 95% CI 1.018-32.565, p=0.048) remained as independent predictors of 1-year mortality in the multivariate analysis (Table 3).

## Discussion

In this retrospective study, we investigated the association between preoperative serum albumin levels and postoperative 1-year mortality in patients undergoing graft replacement of ascending aorta and aortic arch. Preoperative serum albumin level was an independent predictor of 1-year mortality along with diabetes mellitus.

Albumin is a negative acute-phase reactant and its level decreases during injuries and sepsis. Albumin has been reported to be a marker of nutrition and is involved in many biological functions, such as the regulation of oncotic pressure, transport of compounds, and antioxidant activity. 15 Albumin is a well-known marker of nutritional status and has been shown to be related to prognosis. Albumin is a valuable marker of in-hospital malnutrition and frailty. 16 An inadequate nutritional status, indicated by low albumin levels, can lead to poor inflammatory and immune responses to surgery. Hypoalbuminemia has been demonstrated to be associated with postoperative mortality in cardiac and non-cardiac surgeries. 6,8,11,12,17 A previous study conducted in patients with abdominal aortic aneurysms reported that preoperative hypoalbuminemia was associated with increased mortality, longer length of hospital stays, pulmonary complications, and reoperations. 11 Hypoalbuminemia was an independent predictor of mortality in patients with type A and B acute aortic dissection. 12 Serum albumin levels lower than 2.5 g/dL were an independent predictor of mortality and morbidity in patients undergoing cardiac surgery using cardiopulmonary bypass. 17 In accordance with the literature, hypoalbuminemia was an independent predictor of 1-year mortality in patients who underwent graft replacement of ascending aorta and aortic arch in the present study.

The inflammatory pathway is critical for the development of aortic aneurysms. 10,18 T-cell and macrophage infiltrations are present in thoracic aortic aneurysms. 19 Albumin is a well-known negative acute-phase reactant that is reported to decrease during injury and sepsis. Additionally, albumin has an anti-inflammatory effect that can counteract the mechanism of aortic aneurysm formation. 10,20 Another plausible explanation can be the oxidative stress involved in the pathophysiology of aortic aneurysms. 10,18 Thoracic aortic tissues from patients with Marfan syndrome have shown increased levels of oxidative stress. 21 Albumin has also been shown to be involved in extracellular antioxidant defenses. 15 Albumin levels not only decrease in response to inflammation but albumin also has anti-inflammatory and antioxidant effects; therefore, a decrease in albumin levels leads to a decrease in this protective action.

Serum albumin levels are known to be decreased in older patients. 22,23 Similarly, the lower albumin group was significantly older than the higher albumin group in the current study. This age-related decline in albumin levels may be related to frailty and nutritional status. As old age is known to be a predictor of mortality and morbidity in several patient groups undergoing surgery, the influence of age on albumin may have contributed to its role as a predictor of mortality in this study. 24,25

In our study, the ROC curve analysis showed an optimal cut-off value of 4.0, which is higher than that reported in previous studies. A previous study evaluating hypoalbuminemia, mortality, and morbidity in patients undergoing left ventricular device implantation stated that serum albumin < 2.5 g/dL is a risk factor for mortality and morbidity. 26 Other studies reported that serum albumin < 2.5 g/dL is associated with increased mortality and morbidity in cardiac surgeries, including coronary bypass operations or valve surgeries. 17 Another study of patients with acute aortic dissection also showed albumin < 3.4 g/dL as a prognostic factor for mortality. 12 A previous study conducted in patients with abdominal aortic aneurysms undergoing open repair and endovascular aortic aneurysm repair showed that a preoperative albumin ≤ 3.5 g/dL was associated with morbidity and mortality. 11 The higher cut-off value in our study needs further evaluation, but the exclusion of ruptured aneurysm and aortic dissection patients, a relatively younger study population, and a relatively small study sample may have influenced the higher cut-off value. However, our study is among the first to show the cut-off value of serum albumin in patients undergoing graft replacement of ascending aorta and aortic arch.

This study extends prior work by investigating the effects of preoperative albumin levels on patients who underwent graft replacement of ascending aorta and aortic arch, denoting that intervention in the preoperative nutritional status of aortic aneurysm patients may lead to better postoperative outcomes.

This study has some limitations. Firstly, this was a retrospective study, in which inherent biases of confounding and selection existed. Secondly, this was a single-center study that evaluated 183 patients, which is a relatively small sample and the incidence of mortality was not high enough to provide statistical evidence.

## Conclusion

Lower serum albumin levels are associated with higher mortality and morbidity in patients undergoing graft replacement of ascending aorta and aortic arch. This denotes the necessity to routinely measure serum albumin levels before surgery and that the optimization of patients' nutritional status before surgery may be warranted and should be further explored in high-risk patients.

## Figures and Tables

**Table 1:** Baseline characteristics and perioperative data.

	Higher albumin (n=167)	Lower albumin (n=16)	p-value
Demographics			
Female sex	49 (29.3%)	5 (31.3%)	1.000
Age (years)	63.00 (47.00, 71.00)	71.50 (64.00, 77.50)	0.006*
Body mass index (kg/m^2^)	23.73 (21.37, 26.28)	24.06 (20.86, 24.65)	0.297
Comorbidities			
Smoking	76 (45.8%)	7 (43.8%)	0.876
Hypertension	99 (59.3%)	11 (68.8%)	0.460
Diabetes mellitus	14 (8.4%)	3 (18.8%)	0.174
Coronary artery occlusive disease	14 (8.4%)	2 (12.5%)	0.636
Chronic obstructive pulmonary disease	4 (2.4%)	0	1.000
Cerebrovascular disease	11 (6.6%)	1 (6.3%)	1.000
Acute renal failure	0	1 (6.3%)	0.087
Chronic renal failure	6 (3.6%)	3 (18.8%)	0.034*
Liver disease	6 (3.6%)	1 (6.3%)	0.479
Malignancy	1 (0.6%)	1 (6.3%)	0.168
Medications			
β-blockers	52 (31.1%)	7 (43.8%)	0.302
Calcium channel blocker	56 (33.5%)	7 (43.8%)	0.411
Angiotensin receptor blocker	78 (46.7%)	7 (43.8%)	0.821
Statin	53 (31.7%)	6 (37.5%)	0.638
Diuretics	32 (19.2%)	6 (37.5%)	0.105
Preoperative laboratory data			
Alkaline phosphatase (IU/L)	75.00 (63.50, 87.50)	95.50 (65.00, 109.50)	0.083
AST (IU/L)	23.00 (20.00, 28.00)	27.00 (19.50, 44.50)	0.087
ALT (IU/L)	18.00 (14.00, 25.00)	20.50 (13.00, 63.50)	0.318
BUN (mg/dL)	16.10 (13.25, 20.10)	19.50 (13.40, 27.70)	0.185
Creatinine (mg/dL)	0.82 (0.70, 0.99)	0.98 (0.76, 1.18)	0.077
Total bilirubin (mg/dL)	0.70 (0.50, 0.90)	0.70 (0.50, 1.60)	0.399
Total protein (g/dL)	7.10 (6.80, 7.50)	5.90 (5.50, 6.40)	<0.001*
Hemoglobin (g/dL)	13.78±1.79	10.76±1.72	<0.001*
Hematocrit (%)	41.02±5.02	32.21±4.57	<0.001*
WBC (10^3/μL)	6.80 (5.6, 8.11)	8.05 (5.33, 11.11)	0.140
Platelet (10^3/μL)	223.00 (186.00, 269.00)	213.50 (140.50, 270.50)	0.558
Prothrombin time (sec)	0.99 (0.95, 1.05)	1.12 (1.06, 1.24)	<0.001*
C-reactive protein (mg/L)	1.00 (0.40, 2.80)	40.05 (15.05, 78.00)	<0.001*
Operative data			
Emergency	27 (16.3%)	5 (31.3%)	0.164
Anesthesia time (min)	397.28±82.83	390.94±94.64	0.773
Operative time (min)	303.91±87.51	296.13±91.94	0.736
CPB time (min)	165.93±53.72	159.33±51.54	0.649
ACC time (min)	68.00 (45.00, 147.00)	52.00 (36.00, 120.00)	0.262
TCA time (min)	27.00 (0.00, 41.50)	34.50 (21.00, 52.50)	0.244
pRBC transfusion (pack)	1.00 (0.00, 2.00)	4.00 (1.50, 5.00)	<0.001*
FFP transfusion (pack)	5.00 (3.00, 5.00)	5.00 (3.00, 5.00)	0.879
Platelet transfusion (pack)	12.00 (12.00, 15.00)	14.00 (11.00, 15.00)	0.540

Values are presented as the mean ± standard deviation, median (interquartile range) or number of patients (%). AST, aspartate aminotransferase; ALT, alanine aminotransferase; BUN, blood urea nitrogen; WBC, white blood cells; CPB, cardiopulmonary bypass; ACC, aortic cross-clamp; TCA, total circulatory arrest; FFP, fresh frozen plasma; *p < 0.05.

**Table 2:** Postoperative morbidity and mortality.

	Higher albumin (n=167)	Lower albumin (n=16)	p-value
Hospital day (day)	14.00 (11.00, 20.00)	27.00 (22.00, 50.50)	<0.001*
TND	4 (2.4%)	0	1.000
CVA	10 (6.0%)	1 (6.3%)	1.000
Reintubation	6 (3.6%)	2 (12.5%)	0.147
MV>24 hrs	21 (12.6%)	7 (43.8%)	0.004*
RRT	6 (3.6%)	5 (31.3%)	0.001*
AKI	18 (10.8%)	5 (31.3%)	0.034*
Infection	19 (11.4%)	7 (43.8%)	0.003*
Pulmonary Cx	9 (5.4%)	6 (37.5%)	0.001*
MI	1 (0.6%)	1 (6.3%)	0.168
Arrhythmia	19 (11.4%)	3 (18.8%)	0.415
Reoperation	5 (3.0%)	1 (6.3%)	0.427
ICU readmission	11 (6.6%)	3 (18.8%)	0.110
30-d mortality	1 (0.6%)	1 (6.3%)	0.168
1-y mortality	6 (3.6%)	4 (25.0%)	0.006*

Values are presented as the median (interquartile range) or number of patients (%). TND, transient neurological deficit; CVA, cerebrovascular accident; MV, mechanical ventilation; RRT, renal replacement therapy; AKI, acute kidney injury; Pulmonary Cx, pulmonary complication; MI, myocardial infarction; ICU, intensive care unit; *p < 0.05.

**Table 3:** Logistic regression analysis for predictors of 1-year mortality of patients after graft replacement of ascending aorta and aortic arch.

	Univariate OR (CI)	P-value	Multivariate OR (CI)	P-value
Age	1.104 (1.021-1.193)	0.013	1.108 (0.998-1.230)	0.054
Smoking	0.785 (0.214-2.880)	0.715		
Hypertension	2.784 (0.574-13.502)	0.204		
Diabetes mellitus	4.867 (1.132-20.931)	0.033	5.757 (1.018-32.565)	0.048*
Coronary artery occlusive disease	2.839 (0.549-14.681)	0.213		
Chronic renal failure	2.292 (0.258-20.363)	0.457		
Emergency	3.429 (0.908-12.942)	0.069		
Reoperation	3.733 (0.394-35.397)	0.251		
Preoperative hemoglobin	0.634 (0.459-0.876)	0.006	1.050 (0.674-1.636)	0.829
Albumin	0.105 (0.013-0.350)	<0.001	0.116 (0.021-0.641)	0.014*

Values are presented as odds ratio (95% confidential interval). *p < 0.05.
